# Unraveling the phylogenetics of genetically closely related species, *Haemaphysalis japonica* and *Haemaphysalis megaspinosa*, using entire tick mitogenomes and microbiomes

**DOI:** 10.1038/s41598-024-60163-x

**Published:** 2024-04-30

**Authors:** Mohamed Abdallah Mohamed Moustafa, Wessam M. A. Mohamed, Elisha Chatanga, Doaa Naguib, Keita Matsuno, Alexander W. Gofton, Stephen C. Barker, Nariaki Nonaka, Ryo Nakao

**Affiliations:** 1 Department of Entomology, Rutgers School of Environmental and Biological Sciences, Rutgers the State University of New Jersey, New Brunswick, NJ 08901 USA; 2 Laboratory of Parasitology, Department of Disease Control, Faculty of Veterinary Medicine, Hokkaido University, Sapporo, Hokkaido 060-0818 Japan; 3 Department of Animal Medicine, Faculty of Veterinary Medicine, South Valley University, Qena, 83523 Egypt; 4 Department of Biochemistry and Microbiology, Rutgers School of Environmental and Biological Sciences, Rutgers the State University of New Jersey, New Brunswick, NJ 08901 USA; 5 Department of Veterinary Pathobiology, Lilongwe University of Agriculture and Natural Resources, P.O. Box 219, Lilongwe, Malawi; 6 Department of Hygiene and Zoonoses, Faculty of Veterinary Medicine, Mansoura University, Mansoura, 35516 Egypt; 7 One Health Research Center, Hokkaido University, Sapporo, Japan; 8 International Collaboration Unit, International Institute for Zoonosis Control, Hokkaido University, Sapporo, Japan; 9 Division of Risk Analysis and Management, International Institute for Zoonosis Control, Hokkaido University, Sapporo, Japan; 10 Institute for Vaccine Research and Development, HU-IVReD, Hokkaido University, Sapporo, Japan; 11 CSIRO, Health and Biosecurity, Canberra, ACT Australia; 12 Department of Parasitology, School of Chemistry and Molecular Biosciences, The University of Queensland, Brisbane, QLD 4072 Australia

**Keywords:** *Haemaphysalis*, Mitogenome, Microbiome, *Coxiella*, Endosymbiont, Tick, Entomology, Infectious diseases

## Abstract

Ticks have a profound impact on public health. *Haemaphysalis* is one of the most widespread genera in Asia, including Japan. The taxonomy and genetic differentiation of *Haemaphysalis* spp. is challenging. For instance, previous studies struggled to distinguish *Haemaphysalis japonica* and *Haemaphysalis megaspinosa* due to the dearth of nucleotide sequence polymorphisms in widely used barcoding genes. The classification of *H. japonica japonica* and its related sub-species *Haemaphysalis japonica douglasi* or *Haemaphysalis jezoensis* is also confused due to their high morphological similarity and a lack of molecular data that support the current classification. We used mitogenomes and microbiomes of *H. japonica* and *H. megaspinosa* to gain deeper insights into the phylogenetic relationships and genetic divergence between two species. Phylogenetic analyses of concatenated nucleotide sequences of protein-coding genes and ribosomal DNA genes distinguished *H. japonica* and *H. megaspinosa* as monophyletic clades, with further subdivision within the *H. japonica* clade. The 16S rRNA and NAD5 genes were valuable markers for distinguishing *H. japonica* and *H. megaspinosa*. Population genetic structure analyses indicated that genetic variation within populations accounted for a large proportion of the total variation compared to variation between populations. Microbiome analyses revealed differences in alpha and beta diversity between *H. japonica* and *H. megaspinosa*: *H. japonica* had the higher diversity. *Coxiella* sp., a likely endosymbiont, was found in both *Haemaphysalis* species. The abundance profiles of likely endosymbionts, pathogens, and commensals differed between *H. japonica* and *H. megaspinosa*: *H. megaspinosa* was more diverse.

## Introduction

Ticks are arachnids that feed on blood of humans, domestic animals, and wildlife. During the process of blood feeding, ticks can transmit a variety of pathogens including the causative agents of anaplasmosis, ehrlichiosis, borreliosis, piroplasmosis and tick-borne encephalitis^1^. In addition, ticks can cause irritation, allergy and toxicosis^2^. Approximately, nine hundreds of tick species are distributed around the world^3^. However, the taxonomy of ticks remains uncertain due to the limited phylogenetic information on mitochondrial and nuclear genomes^4^. In the field of tick taxonomy and speciation, the classification of closely related tick species often presents a complex challenge. This challenge is not limited to subspecies alone but extends to distinct species with remarkably similar morphological features. Instances of such ambiguity are prevalent among various tick species, where differentiation based solely on visual attributes becomes complex. Notably, the classification of tick species like *Ixodes scapularis* and *Ixodes pacificus*^5,6^, both black-legged ticks inhabiting different regions, underscores this difficulty. Similarly, *Dermacentor variabilis* and *Dermacentor andersoni*^7^, distinguished by their distinct habitats in North America, exhibit analogous morphologies that warrant molecular scrutiny for accurate differentiation. Likewise, species like *Amblyomma maculatum* and *Amblyomma triste*^8^, as well as *Rhipicephalus appendiculatus* and *Rhipicephalus zambeziensis*^9,10^, exemplify the need for genetic techniques to unravel taxonomic uncertainties. Even in Europe, ticks such as *Ixodes hexagonus* and *Ixodes canisuga*^11^ demand molecular methods for accurate identification due to their close resemblance. These cases highlight the importance of molecular, genetic, and morphological approaches in untangling the complexities of tick taxonomy, leading to enhanced comprehension of their ecological roles and disease transmission potential.

A good example for the uncertain taxonomy is the phylogenetic classification of *Haemaphysalis* species, which are predominantly distributed in Asia including Japan*.* The genus *Haemaphysalis* (Koch, 1844)*,* is considered the second largest genus of ticks (166 species) after the genus *Ixodes* (243 species)^12^. Albeit *Haemaphysalis japonica* and *Haemaphysalis megaspinosa* are morphologically distinct from each other, the previous studies could not differentiate between them genetically due to the lack of sequence polymorphisms in the partial sequences of two mitochondrial genes 16S rRNA and NADH dehydrogenase subunit 2 (ND2) genes^13,14^. Moreover, a confusing classification of *H. japonica* and its related species *Haemaphysalis japonica douglasi* and *Haemaphysalis jezoensis* has been debated. *H. japonica* was firstly described by Warburton using male specimens which were collected in Kyushu Island, Japan^15^. The monograph written by Nuttall and Warburton supplemented the information on this species and introduced the description of *H. japonica var. douglasi* using a male specimen collected in northern China^16^ Thereafter, Pomerantzev elevated this variety as a subspecies *H. japonica douglasi*^17^. Meanwhile, *H. jezoensis* was described using specimens collected in Hokkaido Island, Japan by Ogura and Takada^18^. However, *H. jezoensis* was suggested to be morphologically identical to *H. japonica douglasi* from China and Russia and collectively named as *Haemaphysalis douglasi* without taxonomic descriptions^19^. Most recently, *H. douglasi* from Hokkaido and *H. japonica* from Honshu Islands were suggested to be the same species based on comparing the partial sequences of the large subunit ribosomal RNA gene and internal transcribed spacer 2^20^.

Recently, the whole mitochondrial genomes (mitogenomes) are being used to describe the deep phylogenetic structure of ticks^21,22^ and to investigate the demographic patterns and population structure of sympatric tick species^23^. For instance, this approach has yielded a robust phylogenetic hypothesis to resolve *Amblyomma cajennense* species complex through utilizing the mitogenomes for *A. cajennense* s. s., *Amblyomma mixtum*, *Amblyomma tonelliae*, *Amblyomma patinoi*, and *Amblyomma sculptum*^24^. In addition, mitogenome sequencing has become instrumental in uncovering cryptic tick species. For example, in the case of *Rhipicephalus* ticks, sequencing mitogenomes revealed two *Rhipicephalus microplus* clades and ticks presumed to be *R. microplus* from Southern China and Northern India were identified as a cryptic species closely related to *Rhipicephalus annulatus*^25^. Another example is the detection of a potentially novel tick species closely related to *Amblyomma testudinarium* from Myanmar^23^. Simultaneously, the tick microbiome plays a crucial role in tick physiology, host–pathogen interactions, and vector-borne disease transmission^26^. Recent advancements in the field of tick microbiome research have revealed the complex microbial communities harbored by ticks and their potential impact on tick biology and vector competence^27,28^. Tick microbiomes exhibit considerable variation across different tick species^29^. For example, the microbiome of *Ixodes granulatus* is characterized by the presence of Rickettsiales and *Borrelia*^30^, whereas *Haemaphysalis hystricis* predominantly harbors *Acinetobacter* and Rickettsiales^31^. *Haemaphysalis shimoga*, on the other hand, is marked by a significant abundance of *Coxiella* and varying levels of Rickettsiales, *Candidatus* Rhabdochlamydia, and *Stenotrophomonas*^31^. Meanwhile, *Dermacentor steini* displays a unique microbial profile, with prominent *Acinetobacter* and *Francisella*, along with Rickettsiales and Burkholderiales^32^. These examples underscore the distinct microbial communities existing in different tick species, even those closely related, and emphasize the importance of studying tick microbiomes for a comprehensive understanding of tick physiology and their potential roles in disease transmission^31^. Investigating the microbiome of several tick species could provide valuable insights into the development of targeted tick control strategies, potentially disrupting the microbial balance and reducing tick-borne disease burden^33^.

To gain deeper insights into the phylogenetic relationships and genetic divergence between *H. japonica* and *H. megaspinosa*, we employed whole mitogenome sequencing and comprehensive microbiome analysis. These cutting-edge approaches allowed us to elucidate the mitogenomic and microbial characteristics that differentiate these closely related tick species, providing valuable information on their evolutionary history, potential ecological niches.

## Results

### Characteristics of the analyzed mitogenomes

The mitogenomes of 29 *H. japonica* and 18 *H. megaspinosa* specimens from Japan were sequenced: these ranged from 14,678 to 14,683 bp in length. A total of 37 genes were identified, including 13 protein-coding genes (PCGs), 22 transfer RNA (tRNA) genes, and two ribosomal DNA  (rDNA). Additionally, two control regions were observed in the mitogenome. Notably, no gene rearrangements were detected among the 47 newly sequenced *Haemaphysalis* mitogenomes.

The nucleotide sequences of 47 ticks were aligned with the reference mitogenome sequences of *H. japonica* from China (accession number: NC_037246), as well as *Haemaphysalis flava* (accession number: NC_005292). The 29 mitogenomes of *H. japonica* had an average nucleotide identity of 99.4% (99.0–99.9) whereas the 18 mitogenomes of *H. megaspinosa* had an average nucleotide identity of 99.8% (99.4–99.9). On the other hand, the 29 mitogenomes of *H. japonica* had an average nucleotide identity of 99.0% (98.9–99.1) with the 18 mitogenomes of *H. megaspinosa.*

### Phylogenetic relationships inferred from the concatenated sequences of 13 protein-coding genes and two ribosomal DNA genes

**Figure 1:**
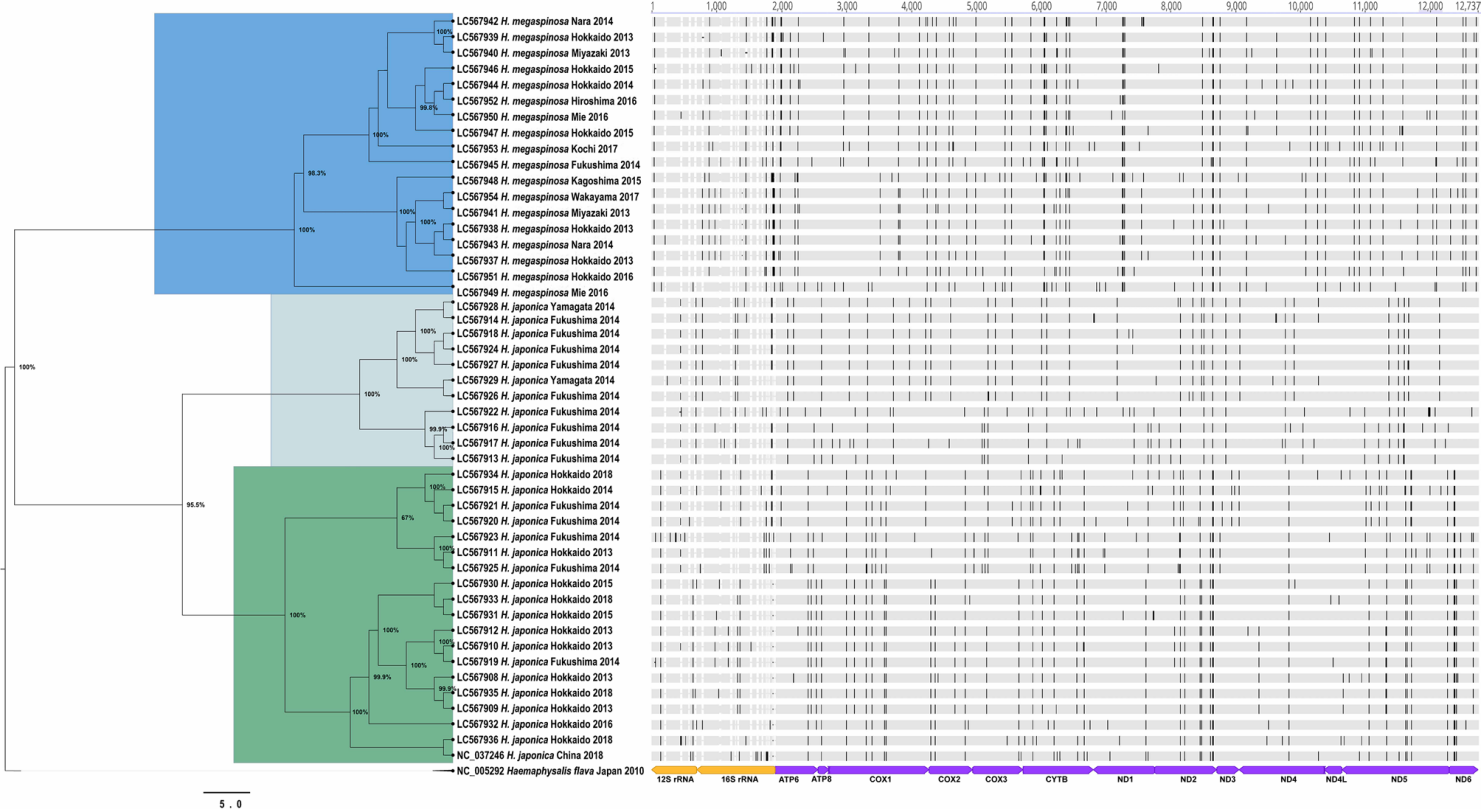
Genetic diversity and phylogenetic relationships of *H. japonica* and *H. megaspinosa* specimens from Japan revealed by Bayesian MCC tree of concatenated of 15 mitochondrial gene sequences. The tree was rooted to *H. flava* which is the putative sister-species to *H. japonica* and *H. megaspinosa* (Barker unpublished data). In the tree, the clade representing specimens from *H. megaspinosa* are highlighted in blue (Hm) while *H. japonica* specimens were subdivided into two clades that are highlighted in light cyan (Hj1) and green (Hj2). The nucleotide differences among the 47 newly sequenced mitogenomes of *Haemaphysalis* collected in Japan were examined. The positions of single nucleotide variations are indicated by vertical lines in the mitogenome sequences.

**Figure 2:**
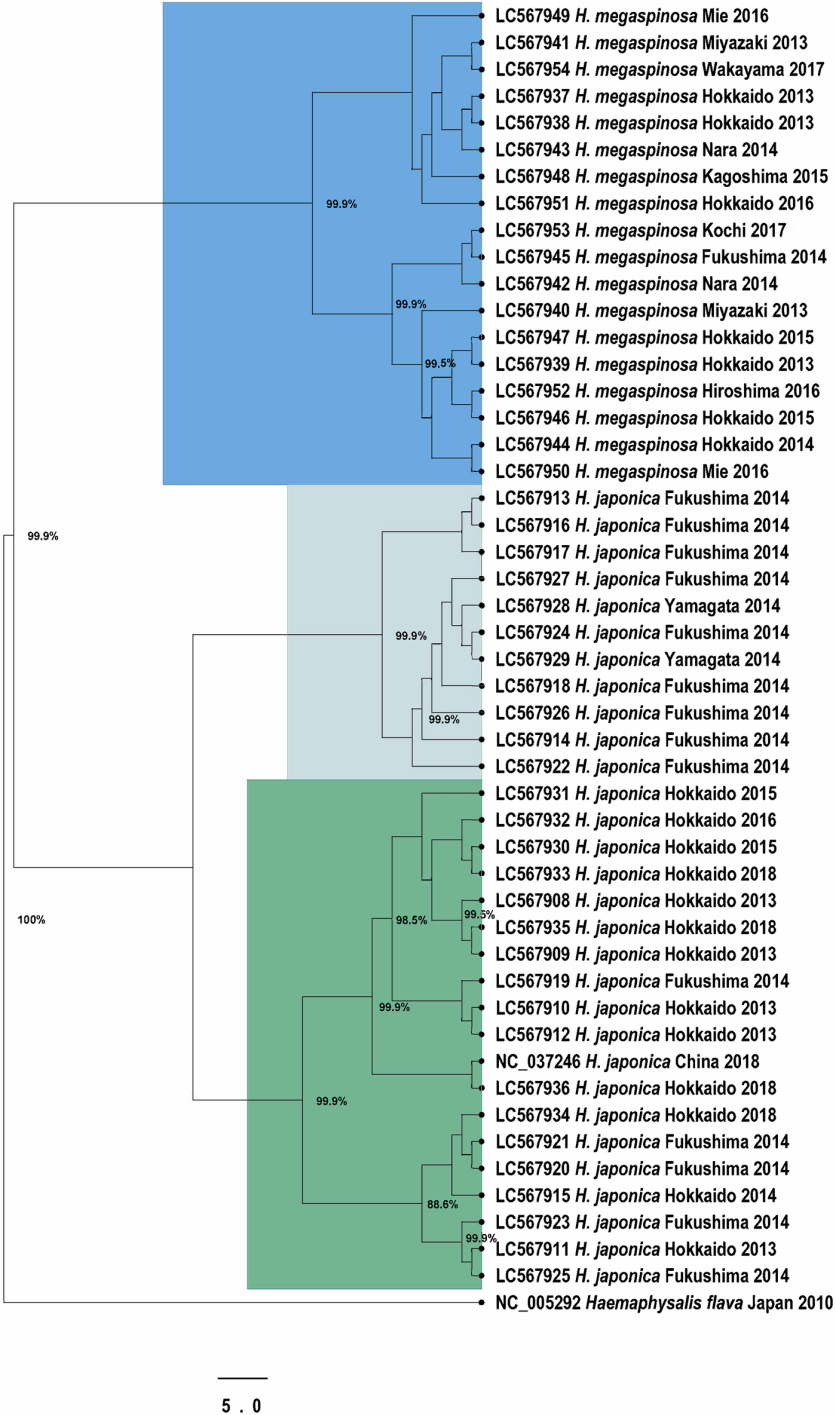
Genetic diversity and phylogenetic relationships among *H. japonica* and *H. megaspinosa* specimens from Japan using a Bayesian MCC tree based on the translated concatenated sequences of 13 protein coding genes. The tree was rooted to *H. flava* which is the putative sister-species to *H. japonica* and *H. megaspinosa* (Barker unpublished data). In the tree, the clade representing specimens from *H. megaspinosa* is highlighted in blue, while *H. japonica* specimens are further subdivided into two clades highlighted in light cyan and green.

The phylogeographic analysis included 29 *H. japonica* and 18 *H. megaspinosa* specimens collected from 10 prefectures in Japan, as well as two reference sequences retrieved from GenBank: *H. japonica* from China (accession number: NC_037246) and *H. flava* (accession number: NC_005292). The maximum clade credibility (MCC) tree topology revealed that the *H. japonica* sequences formed a clade separate from the *H. megaspinosa* sequences (Fig. 1). Furthermore, the *H. japonica* sequences exhibited further subdivision into two additional clades (hereinafter: Hj1 and Hj2). The MCC analysis of the concatenated and translated 13 PCGs further supported the findings of the phylogeographic tree based on the concatenated 15 mitochondrial genes (Fig. 2). To identify suitable markers for molecular differentiation between *H. japonica* and *H. megaspinosa*, we examined the parsimony information sites in 15 mitochondrial genes. Among these genes, the 16S rDNA exhibited the highest number of parsimony information sites, with a total of 30 sites. Additionally, the NAD5 gene showed a substantial number of parsimony information sites, with a total of 37 sites. These markers provide valuable molecular information that can aid in distinguishing between *H. japonica* and *H. megaspinosa* (Supplementary table 1).

### Genetic structure of *H*. *japonica* populations

Our acquired *H. japonica* sequences were grouped into two subclades, Hj1 and Hj2. Hj1 emerged as the prevailing subspecies of *H. japonica* on Honshu Island, while Hj2 dominated Hokkaido Island. Statistical analysis of population genetic structure was performed to assess the influence of geographic factors on the genetic variation within and between populations of *H. japonica*. The results of the Analysis of Molecular Variance (AMOVA) indicated that the genetic structure of *H. japonica* is not influenced by geographic factors, with a lower proportion of genetic variation observed among populations (38.05%) compared to within populations (61.95%). The global *F*_*ST*_ values (*p* < 0.01) indicated significant genetic differentiation within the Fukushima and Yamagata population (*p* = 0.0000) (Table 1).

**Table 1:** Analysis of molecular variance (AMOVA) using the concatenated 15 mitochondrial genes sequences extracted from whole mitogenomes of *Haemaphysalis japonica* populations in Japan.

Source of variation	Degree of freedom	Sum of squares	Variance components	Percentage of variation	*F *_*ST*_
Among populations	1	247.87	15.52	38.05	0.38047***
Within populations	27	682.26	25.27	61.95	

Degree of significance: ***p < 0.0.

### Comparing microbiome diversity between *H*. *megaspinosa* and *H*. *japonica* (Hj1 and Hj2)

After demultiplexing and quality filtering, 1,466,794 high-quality reads were obtained from the Illumina MiSeq sequencer for the microbial analysis, corresponding to 770 features identified through DADA2 quality control analysis. Among the 33 ticks representing two *Haemaphysalis* species, one sample was excluded from the diversity analysis due to the significantly low number of obtained sequences (n = 4123). For the microbial analysis, *H. megaspinosa* was represented by one group (Hm) and *H. japonica* was categorized into two subgroups (Hj1 and Hj2) representing the phylogenetic subclades as illustrated in Fig. 1.

**Figure 3:**
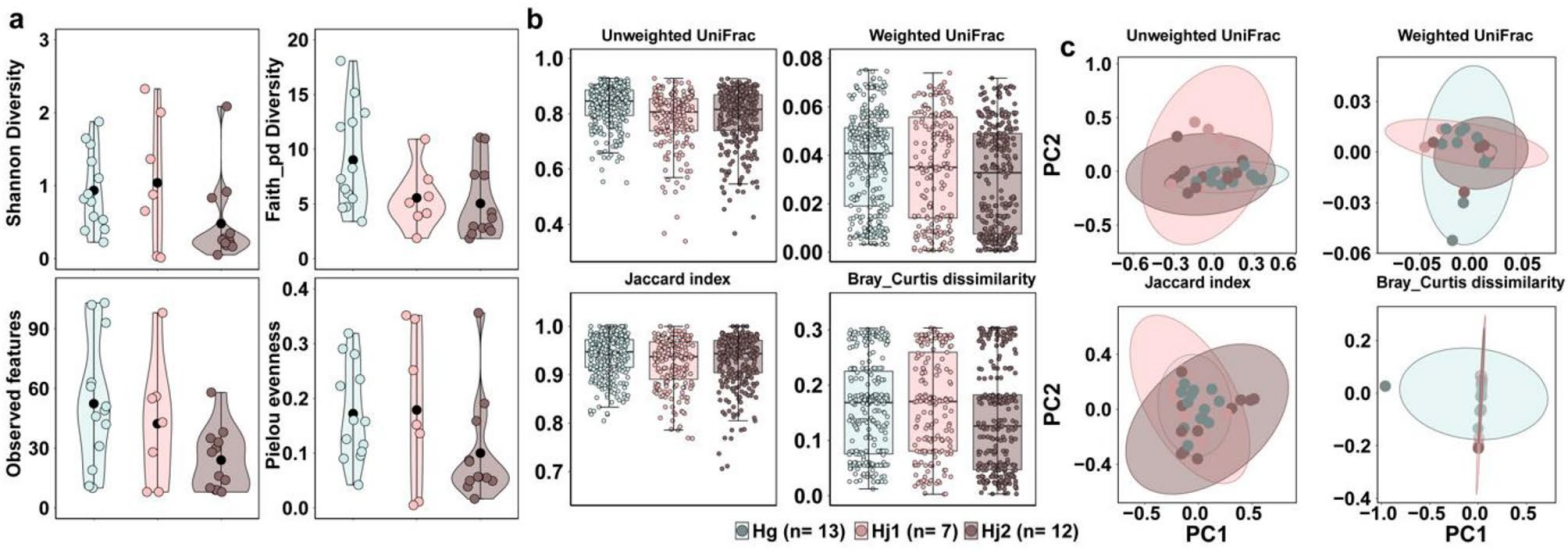
Diversity analyses of microbial populations of *H. japonica *Hj1 (n = 7), Hj2 (n = 12), and *H. megaspinosa* (n = 13) samples. Each dot shows the microbial population from an individual ixodid tick and color represents sample species. (**a**) A box and whisker plot was used to compare the alpha diversity of microbiome communities in *H. japonica* and *H. megaspinosa*. The results showed a significant difference between the two species in terms of Faith PD (*p* < 0.01) and Observed OTUs (*p* < 0.05), but no significant difference was observed for Shannon and Evenness indices (*p* < 0.09 and 0.12, respectively), as determined by the Kruskal–Wallis test. (**b**) The beta diversity of the microbiome in *H. japonica* and *H. megaspinosa* was examined. Pairwise PERMANOVA analysis revealed significant differences in community dissimilarity among the different tick species. Specifically, only Unweighted UniFrac showed a significant difference (*p* < 0.001, pseudo-*F*). (**c**) The PCoA plots, based on Unweighted UniFrac, Weighted UniFrac, Jaccard, and Bray–Curtis dissimilarity metrics, displayed overlapping clusters of samples from each *Haemaphysalis* species.

The Kruskal–Wallis test was employed to determine the significance of alpha diversity between *H. japonica* and *H. megaspinosa*. The results indicated that *H. japonica* exhibited higher alpha diversity compared to *H. megaspinosa*. This was evident through the analysis of various metrics, including Shannon diversity (*p* < 0.09), Faith’s PD (*p*-value < 0.01), observed features (*p*-value < 0.05), and Pielou’s evenness (*p* < 0.12) (Fig. 3a).

The Pairwise Permutational multivariate analysis of variance (PERMANOVA) analysis revealed that beta diversity metrics, including unweighted UniFrac, weighted UniFrac, Jaccard, and Bray–Curtis, differed between *H. japonica* and *H. megaspinosa* (pseudo-*F*, *p* < 0.001, *p* < 0.4, *p* < 0.08, and *p* < 0.2, respectively) (Fig. 3b). The PCoA plots supported the findings from the pairwise PERMANOVA analysis, showing overlapping clusters of samples from each *Haemaphysalis* species (Fig. 3c). Moreover, there was some separation in the clustering of samples based on unweighted UniFrac (Fig. 3c).

**Figure 4:**
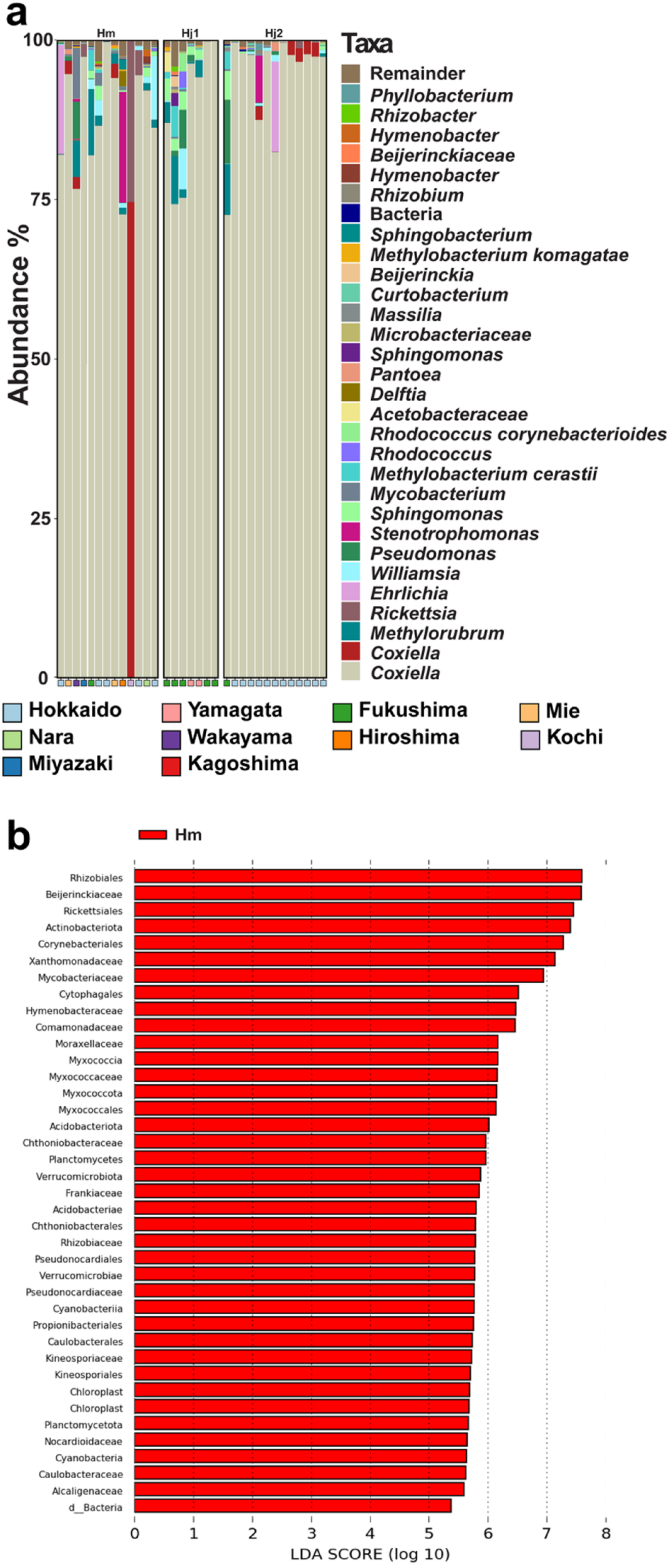
(**a**) The relative abundance (%) of bacterial taxa present in the microbiome of *H. japonica* and *H. megaspinosa*. The top 30 most abundant taxa are shown individually, while the remaining taxa are grouped together. Each bar represents the bacterial taxa identified in a single sample. (**b**) The LEfSe analysis revealed the taxa that were significantly differentially abundant (*p* < 0.05) within *H. megaspinosa*.

The composition of the most abundant bacterial families varied significantly across the different *Haemaphysalis* tick subgroups, as shown in Table 2 and Fig. 4. We provided a comprehensive overview of the total number of reads for each identified bacterial feature in this study. Our findings indicate that every analyzed *Haemaphysalis* species exhibited the presence of *Coxiella* as a predominant potential endosymbiont in their microbiome (Fig. 4a).

**Table 2:** Summary of the most abundant bacterial families in the microbiome of *Haemaphysalis* ticks.

Species	Subclade	Bacterial family	Abundance (%)
*H. megaspinosa*	Hm	*Coxiellaceae*	87.63
		*Rickettsiaceae*	2.43
		*Beijerinckiaceae*	2.28
		*Xanthomonadaceae*	1.40
*H. japonica*	Hj1	*Coxiellaceae*	91.46
		*Beijerinckiaceae*	3.09
		*Sphingomonadaceae*	1.74
		*Nocardiaceae*	1.41
	Hj2	*Coxiellaceae*	86.90
		*Anaplasmataceae*	1.07
		*Xanthomonadaceae*	0.63
		*Beijerinckiaceae*	0.50

By analyzing the relative abundance and conducting linear discriminant analysis effect size (LEfSe) analysis on bacterial genera, we found that the two examined *Haemaphysalis* species displayed similar profiles of potential endosymbionts, specifically *Coxiella* and potential pathogens, specifically *Ehrlichia*. However, they exhibited distinct profiles of commensals, with *H. megaspinosa* samples displaying higher diversity compared to *H. japonica* (Fig. 4b).

## Discussion

The investigation of the phylogeographic structure of tick populations may play a crucial role in enhancing our understanding of tick distribution and the prevalence of tick-borne pathogens^23^. In the present study, we present the first assessment of the genetic diversity and microbiome structure of *H. japonica* and *H. megaspinosa*, which are widely distributed in East and Northeast Asia^34^. Our research stands out as we employed whole mitogenomes of multiple individuals from each tick species to investigate the genetic diversity on a regional scale in Japan. In contrast to earlier studies that struggled to molecularly differentiate *H. japonica* and *H. megaspinosa* or classify *H. japonica* subspecies in the country^13,20^, we successfully analyzed the nucleotide variations in the complete mitogenomes between *H. japonica* and *H. megaspinosa*. Additionally, we examined the intraspecies genetic relationships and population differentiation among *H. japonica* subspecies. Our study benefited from a wide spatial scale of sample collection, enabling us to construct a comprehensive phylogenetic tree using 29 new *H. japonica* and 18 *H. megaspinosa* mitogenome sequences. Intriguingly, the 29 mitogenomes of *H. japonica* were on average only 1% (1.1–0.9%) different to the 18 mitogenomes of *H. megaspinosa* (genetic identity of 99.0% (98.9–99.1). Since Mans et al. (2021) found that pairwise nucleotide differences greater than 5% indicated different species of ticks^35^, the morphology of the voucher specimens of the 29 *H. japonica* and 18 *H. megaspinosa* ticks we studied will be studied in detail, in the future, to test the idea that *H. japonica* and *H. megaspinosa* may be conspecific. Furthermore, we provided valuable insights into the microbiome structure of both tick species based on amplicon sequences obtained from 32 tick samples from Japan.

In our study, tick specimens collected from various biogeographic regions in Japan were examined. Despite the different ecoclimatic habitats, phylogenetic analysis revealed that all sequences from the same *Haemaphysalis* sp. formed a monophyletic group with a high percentile identity of 99.0–99.9%. We found that the 16S rDNA had the highest number of parsimony information sites, totaling 30 sites. Additionally, the NAD5 gene exhibited a significant number of parsimony information sites, with a total of 37 sites. These markers, as presented in Supplementary Table 1, offer valuable molecular information that can aid in the molecular differentiation of *H. japonica* and *H. megaspinosa*. By utilizing these markers, researchers and practitioners can enhance their ability to accurately distinguish between these two tick species, contributing to a better understanding of their distribution and epidemiological significance. Moreover, our analysis revealed that the mitogenomes of *H. japonica* ticks exhibited a biphyletic grouping, with a sequence identity range of 99.0–99.9%. This suggests that *H. japonica* in Japan can be further classified into two subspecies where *H. japonica* found in Honshu Island (Hj1) formed a single clade and *H. japonica* found predominantly in Hokkaido (Hj2) formed the other cluster with the same species from China. This finding supports the previous observation that *H. japonica* (*H. jezoensis* at the time) was morphologically identical to *H. japonica douglasi* from China and Russia^19^.

The statistical analysis of population genetic structure aimed to investigate the genetic differentiation among the Hj1 and Hj2 populations of *H. japonica*. From the MCC tree analysis, it was observed that the Hj2 population consisted of some specimens from Honshu Island. However, no sequences from Hokkaido were found within the Hj1 population. The results of the AMOVA indicated that the genetic structure of *H. japonica* is not significantly influenced by geographic factors, as the proportion of genetic variation among populations (38.05%) was lower compared to within populations (61.95%). This suggests that there is more genetic diversity within each population, and the populations are not highly structured. Despite the clustering of some Hokkaido specimens with Fukushima individuals in the MCC tree, the absence of Hokkaido sequences within the Hj1 population suggests a lack of gene flow or limited genetic exchange between these populations. This may indicate some level of genetic differentiation between the Hj1 and Hj2 populations. The significant genetic differentiation within the Hj1 populations (*p* = 0.0000) further supports the presence of distinct genetic clusters within these regions. It is possible that factors other than geographic proximity, such as local adaptation or genetic drift, are contributing to the observed genetic differentiation.

The comparison of polymorphic sites revealed that relying solely on a single mitochondrial gene for phylogenetic analysis of *Haemaphysalis* spp. may lead to incorrect or incomplete conclusions. Current studies focusing on population structure and phylogenetic analysis of ticks commonly employ multiple mitochondrial genes to draw comprehensive conclusions^36,37^. Moreover, our findings indicate that the *H. megaspinosa* population in Japan exhibits lesser genetic diversity compared to the closely related *H. japonica* population. It is important to note that the evolution of mitochondrial genes can be influenced by various ecological factors, including endosymbionts and host diversity^38^. The disparity in diversity between *H. japonica* and *H. megaspinosa* in Japan may be attributed to variations in endosymbiont genetics that can lead to mitogenome divergence in tick species^39^.

Advancements in sequencing technology and bioinformatic tools have greatly expanded research on vector-associated microbiomes. This progress has significantly contributed to our understanding of tick systematics and the identification of novel tick-borne pathogens^26,40^. Acknowledging the influence of diverse ecological factors and endosymbionts is crucial in understanding the evolutionary dynamics of mitochondrial genes^41,42^. In our study, we conducted a comprehensive investigation of the microbiomes of 32 *Haemaphysalis* ticks, providing valuable insights into the relationship between microbiome composition and species variations. Notably, we observed a predominant presence of a *Coxiella*-like endosymbiont (CLE) within the microbiomes of both *H. japonica* and *H. megaspinosa* ticks. This finding aligns with previous studies that have detected CLE in other *Haemaphysalis* tick species such as *H. longicornis*^43^, *H. shimoga*^31^ and *Haemaphysalis punctata*^44^. The differences in microbiome diversity that we observed between *H. japonica* and *H. megaspinosa* can likely be attributed to various external factors, including the sources and histories of tick blood meals, the presence or absence of pathogenic bacteria and protozoa, and the surrounding environmental conditions^45^. Our study elucidated that the variability among individuals of *H. japonica* and *H. megaspinosa* is primarily driven by the presence of environmental bacteria, as depicted in Fig. 4b. Furthermore, we revealed that both tick species share similar potential pathogens, such as *Ehrlichia* species. This suggests that *H. japonica* and *H. megaspinosa* occupy similar ecological niches and possess comparable physiological characteristics, facilitating the presence of shared potential pathogens.

Our study provides valuable insights into the phylogenetic classification, genetic diversity, and microbiome structure of genetically closely related *Haemaphysalis* species. Through the utilization of complete tick mitogenomes, we successfully differentiated between *H. japonica* and *H. megaspinosa* and examined the intraspecies genetic relationships within *H. japonica*. The identified markers, particularly the 16S rDNA and NAD5 gene, offer valuable tools for accurate molecular differentiation between these tick species. Our findings support the presence of distinct genetic clusters within *H. japonica* populations, with limited gene flow between different regions. The investigation of tick microbiomes revealed the predominance of a CLE and highlighted differences in microbiome diversity between *H. japonica* and *H. megaspinosa*. These findings enhance our understanding of the genetic and ecological characteristics of *Haemaphysalis* ticks and contribute to the knowledge of tick-borne disease epidemiology and control efforts. Further studies exploring the relationship between endosymbiont genetics, mitogenome divergence, and tick microbiomes are warranted to gain a comprehensive understanding of these complex interactions.

## Materials and methods

### Tick samples and DNA extraction

**Figure 5:**
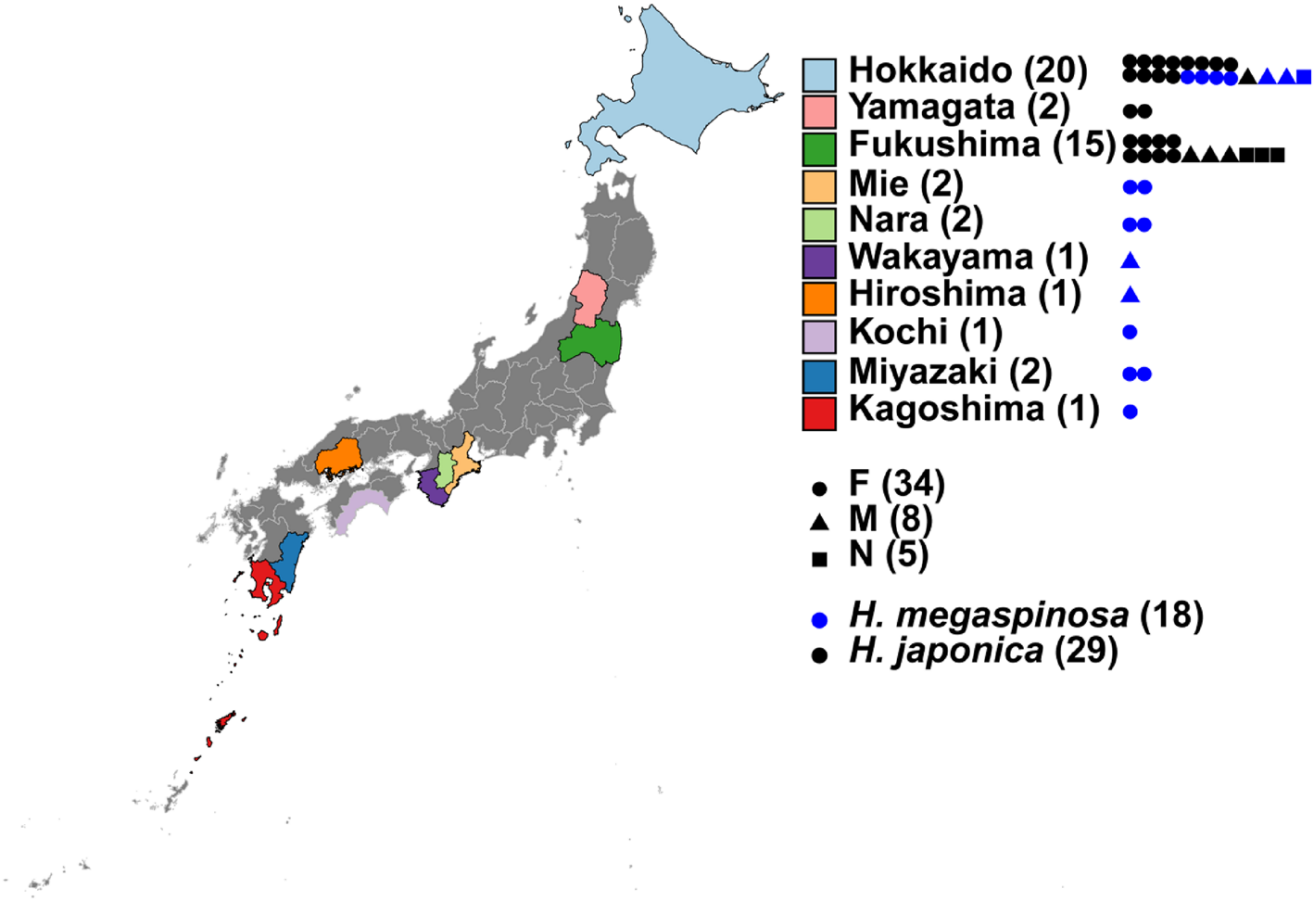
Geographic distribution of *Haemaphysalis* samples used in the present study in Japan. Sample collection sites are illustrated in circles (F: females), triangles (M: males) and squares (N: nymphs). Samples were collected from ten prefectures in Japan.

Ticks were collected from Japan during 2013 to 2018 by flagging a flannel cloth on the plants. The collected ticks were kept in sterile plastic tubes till identification in the laboratory. The morphological identification was conducted by using a stereomicroscope as previously described^46^. A total of 18 *H. megaspinosa* and 29 *H. japonica* (Fig. 5) were morphologically identified and kept separately in Eppendorf tubes at – 20 °C for the molecular analysis (Fig. 5 and Table 3). In the current study, we used the name *H. japonica* regardless of their geographic origin. For DNA extraction, each tick was washed twice with ethanol (70%) and molecular grade phosphate-buffered saline (PBS) and homogenized at 3,000 rpm for 30 s in 100 μL of Dulbecco’s Modified Eagle Medium (DMEM) (Gibco, Life Technologies) by using a Micro Smash MS-100R (TOMY, Tokyo, Japan). A blackPREP Tick DNA/RNA Kit (Analytikjena, Germany) was used to extract DNA from a 50 μL of the tick homogenate^47^.

**Table 3:** Geographic origin, and developmental stage/sex of *Haemaphysalis* ticks.

Sample ID	Tick species	Stage/sex	Prefecture	DDBJ	16S rDNA amplicon
HJ0190	*H. japonica*	Female	Hokkaido	LC567908	
HJ0196	*H. japonica*	Female	Hokkaido	LC567909	Included
HJ0291	*H. japonica*	Female	Hokkaido	LC567910	Included
HJ0292	*H. japonica*	Female	Hokkaido	LC567911	Included
HJ0295	*H. japonica*	Female	Hokkaido	LC567912	Included
HJ0898	*H. japonica*	Female	Fukushima	LC567913	Included
HJ0902	*H. japonica*	Female	Fukushima	LC567914	Included
HJ0945	*H. japonica*	Female	Hokkaido	LC567915	Included
HJ1072	*H. japonica*	Nymph	Fukushima	LC567916	Included
HJ1076	*H. japonica*	Nymph	Fukushima	LC567917	Included
HJ1079	*H. japonica*	Nymph	Fukushima	LC567918	Included
HJ1161	*H. japonica*	Male	Fukushima	LC567919	
HJ1163	*H. japonica*	Male	Fukushima	LC567920	
HJ1164	*H. japonica*	Male	Fukushima	LC567921	Included
HJ1169	*H. japonica*	Female	Fukushima	LC567922	
HJ1170	*H. japonica*	Female	Fukushima	LC567923	
HJ1172	*H. japonica*	Female	Fukushima	LC567924	
HJ1173	*H. japonica*	Female	Fukushima	LC567925	
HJ1174	*H. japonica*	Female	Fukushima	LC567926	
HJ1175	*H. japonica*	Female	Fukushima	LC567927	
HJ1240	*H. japonica*	Female	Yamagata	LC567928	Included
HJ1243	*H. japonica*	Female	Yamagata	LC567929	Included
HJ1463	*H. japonica*	Male	Hokkaido	LC567930	Included
HJ1581	*H. japonica*	Female	Hokkaido	LC567931	Included
HJ2715	*H. japonica*	Female	Hokkaido	LC567932	Included
HJ3546	*H. japonica*	Female	Hokkaido	LC567933	Included
HJ3547	*H. japonica*	Female	Hokkaido	LC567934	Included
HJ3548	*H. japonica*	Female	Hokkaido	LC567935	Included
HJ3549	*H. japonica*	Female	Hokkaido	LC567936	Included
HM0107	*H. megaspinosa*	Male	Hokkaido	LC567937	
HM0110	*H. megaspinosa*	Female	Hokkaido	LC567938	Included
HM0296	*H. megaspinosa*	Female	Hokkaido	LC567939	Included
HM0762	*H. megaspinosa*	Female	Miyazaki	LC567940	
HM0763	*H. megaspinosa*	Female	Miyazaki	LC567941	Included
HM0849	*H. megaspinosa*	Female	Nara	LC567942	Included
HM0862	*H. megaspinosa*	Female	Nara	LC567943	
HM0946	*H. megaspinosa*	Nymph	Hokkaido	LC567944	Included
HM1034	*H. megaspinosa*	Nymph	Fukushima	LC567945	Included
HM1449	*H. megaspinosa*	Male	Hokkaido	LC567946	Included
HM1580	*H. megaspinosa*	Female	Hokkaido	LC567947	Included
HM1725	*H. megaspinosa*	Female	Kagoshima	LC567948	
HM2059	*H. megaspinosa*	Female	Mie	LC567949	Included
HM2060	*H. megaspinosa*	Female	Mie	LC567950	Included
HM2506	*H. megaspinosa*	Female	Hokkaido	LC567951	
HM2629	*H. megaspinosa*	Male	Hiroshima	LC567952	Included
HM2869	*H. megaspinosa*	Female	Kochi	LC567953	Included
HM3469	*H. megaspinosa*	Male	Wakayama	LC567954	Included

### Whole mitogenome sequencing and assembly

Amplification of the complete mitogenomes of *H. megaspinosa* and *H. japonica* was achieved through long- and short-range PCRs, based on a modified protocol from a previous study^22^. The long-range PCR primers, mtG_K23 (5′-TCCTACATGATCTGAGTTYAGACCG-3′) and K26 (5′-ACGGGCGATATGTRCATATTTTAGAGC-3′), along with the short-range PCR primers, H_gap_F1 (5′-YAAYTCCAAAAATTGATGCAAA-3′) and H_gap_R1 (5′-AAGTCAAGRTGCARCAWAAR-3′), were designed by aligning the complete mitogenomes of *Haemaphysalis* genus available in the database. For the long-range PCR, a 50-μl reaction mixture was prepared, consisting of 10 μl of 5 × PrimeSTAR GXL Buffer (Mg^2+^ Plus) (TaKaRa Bio Inc., Shiga, Japan), 4.0 μl of dNTP Mixture (2.5 mM each), 200 nM of each primer, 1.0 μl of PrimeSTAR^®^ GXL DNA Polymerase (TaKaRa Bio Inc.), and 2.0 μl of template DNA. The reaction conditions were set as follows: 45 cycles of denaturation at 98 °C for 10 s, annealing at 60 °C for 15 s, and extension at 68 °C for 10 min. As for the short-range PCR, a 25-μl reaction mixture was prepared, containing 12.5 μl of 2 × Gflex PCR Buffer (Mg^2+^, dNTP plus) (TaKaRa Bio Inc.), 0.5 μl of Tks Gflex DNA Polymerase (1.25 units/μl) (TaKaRa Bio Inc.), 200 nM of each primer, and 1.0 μl of template DNA. The reaction conditions consisted of an initial denaturation step at 94 °C for 60 s, followed by 45 cycles of denaturation at 98 °C for 10 s, annealing at 55 °C for 15 s, extension at 68 °C for 60 s, and a final extension at 68 °C for 5 min. To analyze the amplified PCR products, electrophoresis was performed using a 1.5% agarose gel stained with Gel-Red™ (Biotium, Hayward, CA). The PCR products were then purified using the NucleoSpin Gel and PCR Clean-Up Kit (TaKaRa Bio Inc.).

The long-range and short-range PCR products were combined at equal concentrations, with a ratio of 7:1, respectively. The final DNA concentration of the mixed PCR products from each sample was normalized to 0.2 ng/μl. Subsequently, Illumina sequencing libraries were generated from the purified PCR amplicons using the Nextera DNA Library Prep Kit (Illumina, Hayward, CA). Sequencing was performed on the Illumina MiSeq platform, utilizing the MiSeq reagent kit v3 for 600 cycles. To obtain the complete mitogenome sequences for each *H. megaspinosa* and *H. japonica* sample, the reads were assembled using CLC Genomics Workbench v20.0.4 (Qiagen, Hilden, Germany).

### Comparative analysis of mitogenomes and phylogenetic inference

The complete mitogenome sequences were imported into Geneious version 10.2.6 (Biomatters Ltd., Auckland, New Zealand) and aligned with the mitogenome sequences of *H. flava* (accession number: NC_005292) and *H. japonica* (accession number: NC_037246). Subsequently, 13 PCG sequences and two rDNA sequences were extracted from each mitogenome sequence and concatenated to create a combined alignment. The concatenated sequences of the 15 mitochondrial genes from *H. megaspinosa* and *H. japonica* samples collected in Japan were aligned using the MAFFT software^48^. To determine the appropriate substitution model, PHYML 3.0 software was utilized, employing the Akaike Information Criterion^49^. Furthermore, a Bayesian phylogenetic tree was constructed using BEAST version 1.4, a cross-platform program for Bayesian analysis of molecular sequences through Markov chain Monte Carlo (MCMC) simulations. The GTR nucleotide substitution model with discrete gamma-distributed rate variation was employed to model sequence evolution. Additionally, a strict clock model was selected to assume a constant evolution rate across the entire tree. The Bayesian skyline coalescent model, a demographic model within a Bayesian framework, was used. The MCMC sampling was performed for 50 million generations, with samples collected every 50,000 steps after a burn-in period of 500,000 steps. The MCC tree was determined using TreeAnnotator^50^. The resulting MCC tree was visualized using FigTree version 1.4.4 (http://beast.bio.ed.ac.uk/figtree), with branch lengths proportional to posterior values. Additionally, another tree was constructed using the same Bayesian phylogenetic analysis technique, but this time utilizing the translated sequences of the concatenated 13 PCG sequences. The evolutionary models, MCMC sampling, burn-in period, and visualization methods remained consistent with the previous tree construction.

### Analysis of the genetic structure within *H*. *japonica* populations

Population genetic structure analyses were conducted using the AMOVA implemented in Arlequin software version 3.5.2.2^51^. The genetic variance among and within populations of *H. japonica* collected from Hokkaido Island (n = 13) was compared to Fukushima and Yamagata prefectures (n = 16). The number of permutations was set to 1000, and significance was assessed at a *p*-value threshold of < 0.05 based on the calculated fixation indices (*F*-statistics). *F*_*ST*_, which indicates the degree of differentiation within populations, was used to measure the extent of allelic fixation or identity within populations^52^. *F*_*SC*_, on the other hand, estimated the differentiation among populations within the assigned group. A higher value of *F*_*SC*_ suggests greater heterogeneity among populations. In cases where a strong population genetic structure is present at the analyzed population scale, *F*_*SC*_ is expected to be higher relative to *F*_*ST*_.

### Data deposition and accession numbers

The entire mitogenome sequences of 29 *H. japonica* and 18 *H. megaspinosa* have been submitted to the DNA Data Bank of Japan (http://www.ddbj.nig.ac.jp). The accession numbers for *H. japonica* samples are LC567908-LC567936, and for *H. megaspinosa* samples are LC567937-LC567954.

### MiSeq 16S rDNA amplicon sequencing

A total of 33 genomic DNA samples from *H. japonica* (n = 20) and *H. megaspinosa* (n = 13) (Table 3), along with one DNA extraction blank controls and one negative controls with distilled water, were subjected to PCR amplification targeting the V3-V4 regions of the bacterial 16S rDNA. The Illumina barcoded primers, Illumina_16S_341F and Illumina_16S_805R, were used for the PCR amplification, following the protocols described in references^53,54^. Kapa HiFi HotStart Ready Mix (KAPA Biosystems, Wilmington, MA, USA) was utilized for the PCR reactions. Each PCR reaction consisted of 12.5 μL of 2 × KAPA HiFi HotStart ReadyMix, 5.0 μL of each primer, and 2.5 μL of the tick genomic DNA samples or negative controls. The PCR products were confirmed by agarose gel electrophoresis using a 1.5% agarose gel stained with Gel-RedTM (Biotium, Hayward, CA, USA) and visualized under UV light. The amplicons were purified using AMPure XP beads (Beckman Coulter Life Sciences, IN, USA). Subsequently, libraries were prepared using the Nextera Index Kit (Illumina, San Diego, CA, USA), and sequencing was performed with a MiSeq Reagent Kit v3 (600 cycles) on an Illumina MiSeq instrument following the manufacturer's instructions. The raw sequence data have been deposited in the DNA Data Bank of the Japan Sequence Read Archive under the accession number DRA017340.

### Bioinformatics analysis

The tick microbiome analysis was processed using the obtained sequencing data and the quantitative insights into microbial ecology 2 software (QIIME2) version 2020.2^55^. Initially, the raw sequencing data, obtained from BaseSpace (Illumina), were demultiplexed, quality-checked, and filtered using the q2-demux plugin. The quality-filtered reads were then subjected to denoising using the DADA2 pipeline version 2019.10^56^. The resulting amplicon sequence variants (ASVs) were aligned using the q2-alignment plugin with mafft^48^, and a phylogenetic tree was constructed using the q2-phylogeny plugin with fasttree2^57^. For diversity analysis, a sampling depth of 21,773 reads was selected for comparing the diversity among the examined *Haemaphysalis* tick species. Alpha diversity measures such as Shannon diversity^58^, Faith’s Phylogenetic Diversity (Faith's PD)^59^, observed features^60^, and Pielou's evenness^61^ were calculated. The results were exported and visualized in R using the qiime2R, ggplot2, and phyloseq packages^62^. Beta diversity measures, including unweighted UniFrac distance^63^, weighted UniFrac distance^64^, Jaccard similarity index^65^, and Bray–Curtis dissimilarity^66^, were calculated using QIIME2. The clustering of ASVs according to species was visualized through Principal Coordinates Analysis (PCoA) using the EMPeror plugin in QIIME2^67^ and R. Taxonomic assignment was performed using the q2-feature-classifier plugin^68^ with the classify-sklearn naïve Bayes taxonomy classifier and SILVA classifier reference sequences (release 132). The Decontam package^69^ in R was used to identify likely contaminants introduced during processing. Archaea, eukaryota, potential contaminants, and sequences not assigned to the kingdom level were manually removed for further analysis in QIIME2. A heatmap phylogenetic tree was constructed using the heatmap method in QIIME2^70^. Differential abundance of the 30 most abundant taxonomic groups was visualized using the taxa_heatmap function in the qiime2R package in R. To identify the bacteria contributing to the dissimilarity of the microbiome among tick groups, the LEfSe in the Huttenhower lab Galaxy pipeline^71^ was implemented. Statistical analyses were performed to assess differences in alpha diversities among both *Haemaphysalis* species using a Kruskal–Wallis test. Alpha diversity measures, including Shannon diversity, Faith's PD, observed features, and Pielou's evenness, were considered as response variables, with tick species as fixed effect variables. Subsequently, we examined the impact of species on beta diversity in both *Haemaphysalis* ticks using Adonis PERMANOVA with 999 permutations^72^.

### Supplementary Information

Supplementary Table 1.

## Data Availability

The unprocessed sequence data have been submitted to the DNA Data Bank of the Japan Sequence Read Archive and can be accessed via the DRA accession number DRA017340.
