# Author Correction: Unraveling the phylogenetics of genetically closely related species, *Haemaphysalis japonica* and *Haemaphysalis megaspinosa*, using entire tick mitogenomes and microbiomes

**DOI:** 10.1038/s41598-024-63887-y

**Published:** 2024-06-05

**Authors:** Mohamed Abdallah Mohamed Moustafa, Wessam M. A. Mohamed, Elisha Chatanga, Doaa Naguib, Keita Matsuno, Alexander W. Gofton, Stephen C. Barker, Nariaki Nonaka, Ryo Nakao

**Affiliations:** 1 Department of Entomology, Rutgers School of Environmental and Biological Sciences, Rutgers the State University of New Jersey, New Brunswick, NJ 08901 USA; 2 Laboratory of Parasitology, Department of Disease Control, Faculty of Veterinary Medicine, Hokkaido University, Sapporo, Hokkaido 060-0818 Japan; 3 Department of Animal Medicine, Faculty of Veterinary Medicine, South Valley University, Qena, 83523 Egypt; 4 Department of Biochemistry and Microbiology, Rutgers School of Environmental and Biological Sciences, Rutgers the State University of New Jersey, New Brunswick, NJ 08901 USA; 5 Department of Veterinary Pathobiology, Lilongwe University of Agriculture and Natural Resources, P.O. Box 219, Lilongwe, Malawi; 6 Department of Hygiene and Zoonoses, Faculty of Veterinary Medicine, Mansoura University, Mansoura, 35516 Egypt; 7 One Health Research Center, Hokkaido University, Sapporo, Japan; 8 International Collaboration Unit, International Institute for Zoonosis Control, Hokkaido University, Sapporo, Japan; 9 Division of Risk Analysis and Management, International Institute for Zoonosis Control, Hokkaido University, Sapporo, Japan; 10 Institute for Vaccine Research and Development, HU-IVReD, Hokkaido University, Sapporo, Japan; 11 CSIRO, Health and Biosecurity, Canberra, ACT Australia; 12 Department of Parasitology, School of Chemistry and Molecular Biosciences, The University of Queensland, Brisbane, QLD 4072 Australia

Correction to: *Scientific Reports* 10.1038/s41598-024-60163-x, published online 30 April 2024

The original version of this Article contained an error in the spelling of the author Doaa Naguib which was incorrectly given as Doaa Nagib.

The original Article has been corrected.

